# Supplementary material for: Niche exclusion of a lung pathogen in mice with designed probiotic communities
Source: eLife. 2026 Aug 4;14:RP108304. doi: 10.7554/eLife.108304 (PMC13436956; doi:10.7554/eLife.108304)
Supplement: Peer review history

# Editor report: eLife Assessment

**DOI:** 10.7554/eLife.108304.3.sa0

**Authors:** Bavesh D Kana

**Affiliations:** https://ror.org/03rp50x72 University of the Witwatersrand South Africa

**Keywords:** Solid, Valuable

This study presents a **valuable** framework for the rational design of bacterial probiotics to protect against respiratory infections. The evidence supporting the central claim — that metabolic niche overlap predicts probiotic efficacy — is **solid**, combining innovative in vitro modeling with in vivo validation, though the model appears less effective for probiotics that rely on antimicrobial metabolite production.

# Referee report: Reviewer #2 (Public review)

**DOI:** 10.7554/eLife.108304.3.sa1

Summary:

This study aims to establish a rational framework for designing bacterial probiotics against respiratory infections. The central hypothesis is that in vitro antagonism, particularly through metabolic niche overlap with a pathogen, predicts in vivo efficacy.

Strengths:

(1) Systematic pipeline: The study integrates bacterial isolation, in vitro characterization, model development, and in vivo validation into a cohesive workflow.

(2) Quantitative model: The introduction of the Niche Index (NI) and Niche Index Fraction (NIF) provides a novel, quantitative tool for predicting probiotic efficacy based on ecological principles.

(3) Mechanistic insight: The work dissects different modes of action, clearly demonstrating that inhibition can be driven by specialized metabolite production (CP8) or carbon resource competition (e.g., CP7), with lactate utilization identified as a key factor.

Weaknesses:

(1) Limited model generalizability: The predictive power of the NI model is not universal. It fails to account for the in vivo inefficacy of CP8 (a metabolite-dependent inhibitor) and cannot explain the short-term protection conferred by some non-inhibitory CPs in vivo, suggesting unmodeled mechanisms like immune priming are at play.

(2) Preliminary nature of key findings: The emphasis on lactate consumption as a critical predictor, while interesting, is not sufficiently explored to establish its general importance beyond the specific strains and conditions tested.

Appraisal:

The authors successfully achieve their aim of establishing a rational probiotic-design pipeline. The data robustly support the conclusion that metabolic niche overlap predicts efficacy for many strains, while also clearly delineating the model's limitations, as acknowledged by the authors.

Impact:

This work provides a valuable methodological framework for hypothesis-driven probiotic discovery. The quantitative Niche Index offers immediate utility to the field and, with further refinement, has the potential to become a fundamental tool for developing respiratory therapeutics.

Comments on revised version.

I thank the authors for their meticulous revisions.

# Author comment: Author response

**DOI:** 10.7554/eLife.108304.3.sa2

**Authors:** Kelsey E Hern, Ashlee M Phillips, Catherine M Mageeney, Kelly P Williams, Anupama Sinha, Hans K Carlson, Kunal Poorey, Nicole M Collette, Steven S Branda, Adam P Arkin

**Affiliations:** https://ror.org/01an7q238 University of California, Berkeley Berkeley United States; https://ror.org/041nk4h53 Lawrence Livermore National Laboratory Livermore United States; https://ror.org/058m7ey48 Sandia National Laboratories Livermore United States; https://ror.org/058m7ey48 Sandia National Laboratories Livermore United States; https://ror.org/058m7ey48 Sandia National Laboratories Livermore United States; https://ror.org/02jbv0t02 Lawrence Berkeley National Laboratory Berkeley United States; https://ror.org/058m7ey48 Sandia National Laboratories Livermore United States; https://ror.org/041nk4h53 Lawrence Livermore National Laboratory Livermore United States; https://ror.org/058m7ey48 Sandia National Laboratories Livermore United States; https://ror.org/01an7q238 University of California, Berkeley Berkeley United States

The following is the authors’ response to the original reviews.

**Public Reviews:**

**Reviewer #1 (Public review):**

A summary of what the authors were trying to achieve:

(1) Identify probiotic candidates based on the phylogenetic proximity and their presence in the lower respiratory tract based on phylogenetic analysis and on meta-analysis of 16S rRNA sequencing of mouse lung samples.

(2) Predefine probiotic candidates with overlapping and competing metabolic profiles based on a simple and easy-to-applicable score, taking carbon source use into consideration.

(3) Confirm the functionality of these candidate probiotics in vitro and define their mechanism of action (niche exclusion by either metabolic competition or active antibacterial strategies).

(4) Confirm the probiotic action in vivo.

Strengths:

The authors attempt to go the whole 9 yards from rational choice of phylogenetic close lower respiratory tract probiotics, over in silico modelling of niche index based on use of similar carbon sources with in vitro confirmation, to in vivo competition experiments in mice.

Weaknesses:

(1) The use of a carbon source is defined as growth to OD600 two SD above the blank level. While allowing a clear cutoff, this procedure does not take into account larger differences in the preferences of carbon sources between the pathogen and the probiotic candidate. If the pathogen is much better at taking up and processing a carbon source, the competition by the probiotic might be biologically irrelevant.

While the definition of carbon utilization in this work is a commonly used definition, we agree that there are numerous ways that one could define carbon utilization. We also agree that it is possible that inclusion of additional features of carbon consumption such as the order of prioritization of carbon sources by CP could improve the model. Our data in Figure 3H and 3I do suggest that certain carbon sources may be disproportionately important for predicting antagonistic phenotypes. However, given that the objective of this work was to develop a simple model to aid in the design of probiotic communities, we feel that the current definition of carbon utilization allows maximum accessibility and is suitable for our needs. Work is currently underway to identify additional features, such as carbon source processing efficiency, that may improve the model’s utility.

(2) The authors do not take into account the growth of candidate probiotics in the presence of Bt. In monoculture, three of the four most potent candidate probiotics grow to comparable levels as Bt in LSM.

Yes, our model only accounts for a one way interaction (effect of pathogen on CP). This is for two reasons (1) We are only interested in characterizing and modeling the antagonistic potential of the CP on the pathogen as this antagonism, we propose, is what gives a CP therapeutic potential (2) The degree to which co-culture with Bt impacts CP activity will be captured by the performed competition experiments and therefore any inhibition of the CP by Bt will be accounted for.

While further investigation of the effect of Bt on the growth of the CP may not be necessary to achieve our objective, we agree that ecologically it would be interesting to understand this dynamic better. To explore this, we conducted co-culturing studies between each CP and Bt or media-only control and measured the amount of CP after 24 hours of co-culture. From the data it appears that only a small number of organisms (CP4, CP7 and CP19) are significantly inhibited by the pathogen at the 1:1 ratio tested. This result is perhaps unsurprising as these CPs have the highest niche index and therefore have a greater metabolic overlap with the pathogen.

These data have been incorporated into Figure S1B and additional text has been added to line 157 and the methods at line 712.

(3) Niche exclusion in vivo is not shown. Mortality of hosts after infection with Bt is not a measure for competition of CP with the pathogen. Only Bt titers would prove a competitive effect. For CP17, less than half of the mice were actually colonized, but still, there is 100% protection. Activation of the host immune system would explain this and has to be excluded as an alternative reason for improved host survival.

We have revised the manuscript to address these issues as follows:

(1) We include Bt titer data as suggested, displayed in a new figure (Figure 5F). The results indicate that CP8 fails to reduce Bt titers as compared to the no-CP control, whereas the other CPs tested (CP13, CP17, CP19, CP20, and CP26) do reduce Bt titers to statistically significant degrees (p-value < 0.05 by ANOVA/Tukey). These results support the idea that the CPs competitively exclude Bt in vivo (as they do in vitro), with the notable exception of CP8 (which competitively excludes in vitro but not in vivo, consistent with the mortality results). Further, additional spearman correlation analysis was performed to understand the relationship between the Niche Index value for a given CP and pathogen instantiation when pre-treatment with a given CP is performed. We found that there was a strong relationship between NI_CP_ and pathogen load (r = -0.84, P<0.0001, 95% CI [-0.90 to -0.76], N = 77) such that prophylactic treatment with a CP with a high Niche Index value strongly correlated with lower pathogen load following Bt challenge. Text describing these findings has been added at line 471.

(2) We include survival studies of mice prophylactically treated with non-viable CPs, displayed in a new figure (Figure S7). Viability is required for niche exclusion, so protection conferred by non-viable CPs must be due to other effects such as elicited immune responses. We found that non-viable CPs provide some protection when administered at 3 days prior to *Bt* challenge, though not to the same degree as viable CPs. Together, our data suggest that with the day 3 dosing schedule there are alternative mechanisms of protection (potentially including immune priming) that our current model does not capture. These results are described in further detail at line 460.

Appraisal:

(1) Based on phylogenetic comparison and published resources on lower respiratory tract colonizing bacteria, the authors find a reasonably good number of candidate probiotics that grow in LSM and successfully compete with the pathogenic target bacterium Bt in vitro.

(2) In vivo, only host survival was tested, and a direct competition of CP with Bt by testing for Bt titers was not shown.

Impact:

Niche exclusion based on competition for environmentally provided metabolites is not a new concept and was experimentally tested, e.g. in the intestine. The authors show here that this concept could be translated into the resource-poor environment of the respiratory tract. It remains to be tested if the LSM growth-based competition data in vitro can be translated into niche exclusion in vivo.

**Reviewer #2 (Public review):**

Summary:

This study aims to establish a rational framework for designing bacterial probiotics against respiratory infections. The central hypothesis is that in vitro antagonism, particularly through metabolic niche overlap with a pathogen, predicts in vivo efficacy.

Strengths:

(1) Systematic pipeline: The study integrates bacterial isolation, in vitro characterization, model development, and in vivo validation into a cohesive workflow.

(2) Quantitative model: The introduction of the Niche Index (NI) and Niche Index Fraction (NIF) provides a novel, quantitative tool for predicting probiotic efficacy based on ecological principles.

(3) Mechanistic insight: The work dissects different modes of action, clearly demonstrating that inhibition can be driven by specialized metabolite production (CP8) or carbon resource competition (e.g., CP7), with lactate utilization identified as a key factor.

Weaknesses:

(1) Limited model generalizability: The predictive power of the NI model is not universal. It fails to account for the in vivo inefficacy of CP8 (a metabolite-dependent inhibitor) and cannot explain the short-term protection conferred by some non-inhibitory CPs in vivo, suggesting unmodeled mechanisms like immune priming are at play.

The NI model is not able to identify antagonism of metabolite-dependent inhibitors as their inhibitory activity is unrelated to the variables for which the model accounts. Based on the NI model, CP8 is predicted to have the least metabolic overlap with the pathogen which may explain its in vivo inefficacy. We do agree that short-term protection is only moderately related to NI (r = 0.48, P<0.0001, 95% CI [0.33 to 0.62], N = 115) and may represent an unmodeled alternative mechanism of protection as discussed at line 445, 466 and 523. We have added additional data in Figure S6 and corresponding text at line 444 which gives additional information about CP8 colonization in the context of infection.

(2) Preliminary nature of key findings: The emphasis on lactate consumption as a critical predictor, while interesting, is not sufficiently explored to establish its general importance beyond the specific strains and conditions tested.

Indeed, our model and assertions about critical predictors of antagonism only extend to the specific strains and conditions tested. While we cannot assert that lactate consumption is a critical predictor of antagonism universally, several other studies have indicated the importance of lactate in infection at other body sites [53-57].

To further characterize the role of lactate utilization in the respiratory context, we performed an ex vivo experiment to measure lactate concentrations in respiratory tissue with or without treatment with a key isolate - CP19. After 24 hours of incubation, we found that lactate levels were significantly reduced in the CP19-containing homogenate compared to the PBS-only control (Figure S8A). Additionally, the pathogen was unable to grow in the CP19 conditioned homogenate but was able to grow in the untreated homogenate (Figure S8B). This indicates that CP19 can deplete the total lactate in lung tissue, and that this conditioning can inhibit pathogen growth in the lung tissue. These results are reported in a new supplementary figure (Figure S8) and summarized in corresponding text (line 485), with a description of the experimental procedure in the Methods section (line 924). While this does not prove our theory about the importance of lactate utilization universally, we believe that our work contributes to the growing body of evidence around lactate and its role in infection. Work is ongoing to expand the number of strains screened and determine the generalizability of particular carbon sources and their role in interbacterial antagonism.

Appraisal:

The authors successfully achieve their aim of establishing a rational probiotic-design pipeline. The data robustly support the conclusion that metabolic niche overlap predicts efficacy for many strains, while also clearly delineating the model's limitations, as acknowledged by the authors.

Impact:

This work provides a valuable methodological framework for hypothesis-driven probiotic discovery. The quantitative Niche Index offers immediate utility to the field and, with further refinement, has the potential to become a fundamental tool for developing respiratory therapeutics.

**Recommendations for the authors:**

**Reviewer #1 (Recommendations for the authors):**

Suggestions for improved or additional experiments, data or analyses.

(1) CP titers at the end of the coculture experiment are missing in LSM.

To quantify pathogen abundance after co-culture, cultures were plated on carbenicillin-100 to select for only colonies of the pathogen. As a result, no data about CP abundances were collected in the original experiments. However, we agree that ecologically it would be interesting to understand this dynamic better. We have added additional data about the impact of the pathogen on CP in co-culture to Figure S1B.

(2) Bt titers in mice are essential to claim niche exclusion happens in vivo, and immune-mediated effects have to be excluded.

Please see response to question 3 of the public review.

(3) The definition of the use of carbon sources should be refined. Qualitative differences between the pathogen and the CP with regard to the usage of a given carbon source might have a substantial impact on the actual competitive effect.

The definition of carbon utilization is stated at line 811. While we agree that there may be other carbon-consumption related variables (rate of growth on a particular carbon source, amount of biomass generation on that carbon source etc) that could be used in the model, for the purposes of this study a binary (can versus cannot grow on the carbon source) was sufficient. Work is currently ongoing to determine if metrics of growth on carbon sources such as those listed would improve the predictive capability of the model.

**Reviewer #2 (Recommendations for the authors):**

(1) Experimental & Analytical Suggestions:

(a) To further validate the role of lactate, consider measuring lactate concentration in the airways of mice colonized by key CPs (e.g., CP7, CP19) versus controls. This would directly test if in vivo protection correlates with local lactate depletion.

Unfortunately due to the funding for this project ending, we weren’t able to perform additional animal experiments. However, we were still able to test lactate utilization by CP19 in the respiratory context via an ex vivo experiment. We inoculated mouse lung homogenates with 10^6^ CFU of CP19, or PBS as a negative control, and co-incubated for 24 hours. After 24 hours, we measured lactate levels and found that they were significantly reduced in the CP19-containing homogenate compared to the PBS-only control (A). Additionally, we measured the growth of the pathogen in CP19 conditioned (+CP19) and untreated (-CP19) homogenates and found that the pathogen was unable to grow in the CP19 conditioned tissues (B). This indicates that CP19 can deplete the total lactate in lung tissue, and that this conditioning can inhibit pathogen growth in the lung tissue. These results are reported in a new supplementary figure (Figure S8) and summarized in corresponding text (line 485), with a description of the experimental procedure in the Methods section (line 924).

(b) The finding that CP8 provides no in vivo protection despite in vitro efficacy warrants further investigation. We suggest quantifying CP8 and Bt loads in co-colonized mice to determine if the probiotic fails to persist during infection or if the pathogen evades inhibition.

Please see updated Figure S6 and accompanying text at line 444.

(2) Quantitative Analysis:

Please consider adding a brief justification in the manuscript explaining why the specific Niche Index formula (based on electron equivalents of shared carbon sources) was selected over alternative ecological metrics for quantifying niche overlap.

Text was added starting at line 264 explaining our reasoning for choosing this model.
